# Adherence to adjuvant endocrine therapy among breast cancer survivors: a systematic review and meta-synthesis of the qualitative literature using grounded theory

**DOI:** 10.1007/s00520-020-05585-9

**Published:** 2020-06-29

**Authors:** Othman AlOmeir, Nilesh Patel, Parastou Donyai

**Affiliations:** 1 School of Pharmacy, University of Reading, Whiteknights Campus, Reading, UK; 2 Department of Pharmacy, University of Reading, PO Box 226, Whiteknights, Reading, Berkshire RG6 6AP UK

**Keywords:** Adherence, antineoplastic agents, hormonal, Systematic review, Grounded theory, Qualitative research, Breast neoplasms

## Abstract

**Purpose:**

Numerous studies have examined non-adherence to adjuvant endocrine therapy in women recovering from breast cancer, but none provides a comprehensive theory to explain the challenges of long-term medication taking and resilience needed to continue. The aim of this study was to source, appraise, and synthesize data from existing qualitative studies to develop an in-depth explanatory model of non-adherence and discontinuation of hormonal medication among breast cancer survivors.

**Methods:**

A comprehensive search of databases and the literature identified 24 eligible qualitative studies published 2010–2019. Quotations (*n* = 801) listed within these papers and the original author interpretations were synthesized using NVivo, and grounded theory methodology.

**Results:**

At the beginning, knowledge about adjuvant endocrine therapy, trust in doctors, and worries and expectations, mean agreeing to medication is the only viable option, akin to a Hobson’s choice. Thereafter, women’s ability to deal with medication side-effects, knowledge and support received affect their decision to continue, akin to a horned dilemma where giving up the medication risks cancer recurrence and continuing means reduced contentment. Women stopping medication altogether question treatment necessity, search for normalcy and prioritize quality of life.

**Conclusion:**

Shared experiences and understandings were uncovered by examining commonalities in existing publications. The core category explained the difficulties women face with the initial decision to accept long-term endocrine therapy and then the everyday challenges of continuing or deciding to stop treatment early. An educational tool to inform survivors and health professionals about these challenges could potentially improve women’s experience on treatment and in turn their adherence.

**Electronic supplementary material:**

The online version of this article (10.1007/s00520-020-05585-9) contains supplementary material, which is available to authorized users.

## Background

Breast cancer is the most common type of cancer in women across the world, albeit with a lower incidence in most of the developing regions (age-standardized at below 50 per 100,000), compared with Western Europe (92.6 per 100,000) or North America (84.8 per 100,000) [1]. However, improvements in screening, early diagnosis and treatment in higher-income regions have resulted in a continued fall in death rates from breast cancer in some countries such that the 2019 annual breast cancer age-standardized death rate in North America was estimated to be 12.6 per 100,000 and in the EU 13.36 per 100,000 [2]. For comparison, this mortality statistic is 15.5 per 100,000 in Central and Eastern Europe, 18.4 in Northern Africa, and 21.6 in Polynesia [1]. Improvements in treatment include the use of long-term endocrine therapy (ET) in hormone receptor (HR) positive cases, which account for two-thirds of all breast cancer diagnoses [3]. Prescribing ET, i.e. tamoxifen or an aromatase inhibitor to take by mouth, as adjuvant treatment after surgery or radiation therapy reduces cancer recurrence and mortality rates [4, 5]. However, research consistently shows challenges for some women to fully adhere to their course of ET [6].

Adherence is defined as ‘the extent to which a person’s behaviour corresponds with agreed recommendations from a health care provider’ [7]. According to guidelines, ET as adjuvant treatment for breast cancer should be taken for at least 5 years [8] with studies showing even better results when used for 10 years [9, 10]. However, the literature shows variation in adherence to ET in breast cancer, with studies reporting adherence to range from 41 to 72%, with early discontinuation of 5-year courses being 31 to 73% [11]. Rather than being static, it appears that adherence to ET decreases over time going from 90% in the first year to 77% in the third and 51% in the fifth years [12]. This is despite knowing that low adherence to ET is associated with an increase in all-cause mortality [13], while longer adherence periods lower the risk of mortality and recurrence [12]. Thus non-adherence to ET, like in many other conditions, appears to be a complex problem which is worthy of further exploration with in-depth, interpretive approaches [14].

Although multiple qualitative studies have been undertaken to understand and describe women’s perceptions and experiences of a breast cancer diagnosis and its treatment in different global settings, none provides a comprehensive theory to explain all the challenges of long-term medication-taking in HR-positive cases and the resilience needed to continue. In addition, no secondary research has collected, compared and analysed these different studies to develop an all-encompassing theoretical insight of a phenomenon which may well have experiential universality, justifying a review of the global literature. The purpose of this qualitative systematic review and meta-synthesis then was to source, appraise and synthesize data from existing qualitative studies to develop an in-depth explanatory model of non-adherence with ET in breast cancer survivorship across the world. The aim was to use a grounded theory (GT) and an interpretivist approach. GT was chosen as the method of analysis so that the findings could meaningfully feed into the next phase of investigations; an interview study which itself aimed to use GT. GT has its roots in sociology and is based on the notion that concepts grounded in the data can be examined and integrated into a core category [15]. The interpretivist approach is concerned with deconstructing the meanings of the phenomenon being researched in order to explain why it operates the way it does [16].

## Methods

### Data retrieval, abstraction and appraisal

This was a systematic review and meta-synthesis of the qualitative literature using grounded theory. A comprehensive search of the published literature was completed via multiple relevant databases to identify qualitative research papers on the topic of women’s adherence to oral ET in breast cancer, namely, PubMed, Web of Science, Cumulative Index to Nursing and Allied Health Literature (CINAHL), PsycINFO, Wiley Online Library and ProQuest. In addition, Google scholar, Taylor & Francis online, ScienceDirect and SpringerLink were searched to identify studies that may not have been indexed in the previous databases. The references in the identified articles were also scanned for relevant studies that may have been missed in the database searches. The search was conducted by the first author (OA) and verified by the third (PD). The searching began on 01/11/2017 and a final check took place on 23/01/2020.

Articles were included if they were primary research studies, used qualitative methodology, investigated adherence to adjuvant ET in the management of HR-positive breast cancer and were written in the English language. We included papers published in the decade 2010–2019 reasoning this era would provide data most relevant to modern practice. Studies were excluded if not written in English, used quantitative methodology, were reviews, were not specifically about breast cancer or did not investigate adherence to ET. A full search history is detailed in the Online Resources 1, 2, 3, 4, 5, 6, showing the construction of the search queries, the narrowing down of the searches and the final yield from each database. The articles yielded from each database were then reviewed by the first author (OA) who read the titles and abstracts of each and collected all potentially relevant articles for discussion with the third author (PD). Both authors applied the inclusion and exclusion criteria to this pool of potentially eligible articles to finalize the list of included articles. A grid was created to summarize the 24 included studies (Online Resource 7).

Study quality was evaluated by the first author (OA) and compared with an independent evaluation by the second (NP) (with a discussion to resolve any differences) using criteria based on the work of Hawker et al. [17]. The appraisal involved using nine items on a checklist to assess the quality of different elements of each paper, rating them as good (g), fair (f), poor (p) or very poor (vp) (Online Resource 8). The inter-rater reliability was not recorded, but there were less than 10 minor disagreements (i.e. where the criteria selected were in close agreement—e.g. good vs fair) between the evaluators from a total of 216 possible ratings. Hawker et al.’s [17] tool was designed to provide insight into study strengths and weaknesses rather than offering a cut-off for exclusion of evaluated studies. The quality of the studies included in this review varied but none was deemed to be of poor quality that would render their findings meaningless. This paper’s compliance with the ‘Enhancing transparency in reporting the synthesis of qualitative research’ (ENTREQ) criteria is shown in Online Resource 9.

### Data analysis

The first author (OA) completed data analysis in consultation with the third (PD) who provided guidance and supervision. Despite publications covering the topic generally [18, 19], on closer scrutiny, no guidance detailed *how* to undertake a GT synthesis of data using existing publications as the data source. The authors therefore shaped their own methodology, drawing heavily on the work of Atkins et al. (2008) [20] for the data extraction elements and using previous GT expertise for the detailed data analysis [21, 22].

The aim was to deconstruct the findings of the retrieved studies in order to reconstruct them within a GT framework. To do this, using the method of Atkins (2008), the studies were read in detail first. Then all of the participant quotes evidenced within the original studies (including those from supplementary materials and appendices) from women relaying their experiences with ET following the initial treatment of their breast cancer were identified. Then every one of these quotes were extracted to form a new dataset of quotes (within the retrieved studies) using the software MindManager® (v. 2018). This software was chosen because each page allows a limitless set of quotes to be displayed, minimized and rearranged in interconnected or distinct mind-maps.

The analysis began by identifying first order constructs relating to these quotes. Our approach was to firstly deconstruct the findings of the retrieved studies within a grounded theory (GT) framework. To do this, we devised a step-by-step plan on how to organize and deal with the dataset. Thus the quotes were organized along a treatment timeline in MindManager®, to span experiences from receiving the initial prescription, to treatment continuation and to treatment cessation. Ambiguous quotes were excluded. An example of an ambiguous quote is “No one but God will discuss death with me. Only God cares for my spirit.”, because it could not be associated directly with a specific element of the treatment. The process of analysis was to interpret each quote, making detailed memos and asking questions such as _“what is going on here?”, “Why?”, “How?” and “Where?”, to generate first-order concepts for all the quotes. These concepts were then grouped under higher-level criteria accompanied by writing detailed criteria for each category. To minimize the impact of preconceptions, the researcher (OA) wrote his own interpretation of each quote without reference to the original paper. He then compared his interpretations against the original authors’ which offered further explanation and context. This allowed the creation of novel constructs that were informed by interpretations made by the original authors, creating new superordinate groupings.

Quotations and interpretations were then transferred to the NVivo software (v11) and further analyzed using open, axial and selective coding in line with GT methodology to develop the categories [15]. This involved brainstorming, questioning the data, making constant comparisons (which contained some deductive elements), thinking reflectively and using inductive coding, as well as making diagrams to show the links and the flow of the process under investigation. A paradigm model was used which was composed of causal conditions (the circumstances or events resulting in the phenomenon), actions/interactions (activities directed toward the phenomenon), consequences (the outcome of the actions/interactions) and mediating factors (the conditions that affect the actions/interactions). These were mapped for each of the categories by posing statements in the form of “If *this* happens, I do *this* in the anticipation that *this* will happen”. Finally, an overarching theoretical scheme was created to interrelate the categories within one core category to conceptually encompass and explain the collective experiences of the participants of the retrieved studies. The measures taken to ensure rigour and trustworthiness included prolonged engagement with the data, description of the study procedures and detailed audit trails.

## Results

**Fig. 1:**
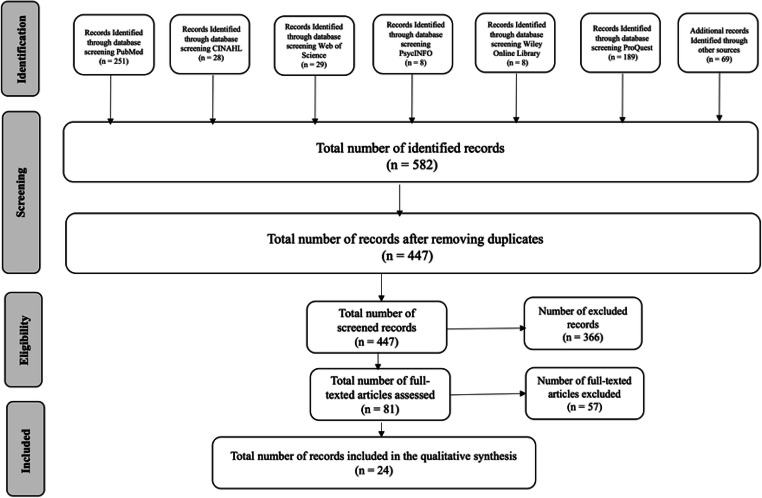
Database searches and article retrieval. *Note.* 366 records were excluded at screening and 57 records at full-text analysis because they were not primary studies, did not use qualitative methods or did not provide data on hormonal therapy in breast cancer.

**Fig. 2:**
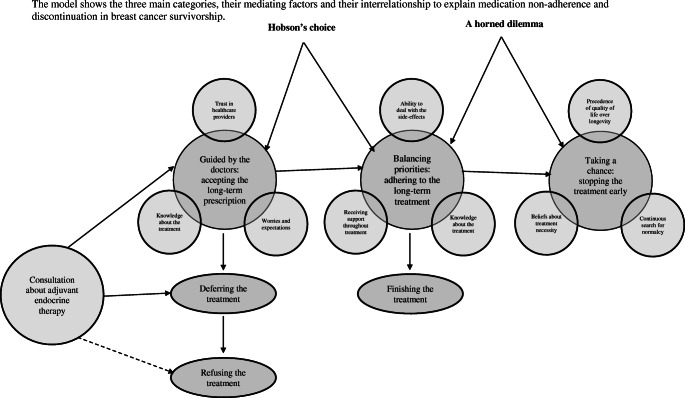
Representation of the core category ‘Hobson’s choice or a horned dilemma’

A total number of 582 papers were first identified narrowed to 447 with duplicates removed. The titles and abstracts of these 447 papers were reviewed for eligibility. A total of 81 papers were included in the full-texted assessment, from which 57 papers were excluded, meaning 24 articles were selected for qualitative synthesis (Fig. 1). There were a total of 801 quotes, with 169 for the phase receiving the initial prescription, 506 for treatment continuation (*n* = 506) and 87 for treatment cessation, with 39 ambiguous quotes. Three main categories, explaining women’s adherence to ET in breast cancer survivorship as conceived by the participants of the studies included in the meta-synthesis are described below. The core category ‘Hobson’s choice or a horned dilemma?’ encapsulates the findings and provides an all-encompassing GT of the challenges of long-term medication taking and the resilience needed to continue (See Fig. 2). Hobson’s choice depicts a ‘free choice’ where in reality only one option is offered without any alternatives [23]; a horned dilemma is about facing two equally problematic options whereby choosing either leaves you, metaphorically, impaled by your own decision [24].

### Core category ‘Hobson’s choice or a horned dilemma?’

At the start of treatment, when women sign up to long-term ET, they do so without feeling that they have a real choice. They are concerned about the recurrence of cancer, and believe in the necessity of the treatment, leaving them with no basis to decline. This is akin to Hobson’s choice—there is no viable alternative. This interpretation of the situation helps many women continue to take the medication for the full duration of treatment. On the other hand, some women think of adhering to their ET as a horned dilemma. They fear the treatment and its potential side-effects or have experienced the severe side-effects, leaving them confronted with two equally bad options. There is the choice to continue but experience the side-effects and there is the choice to stop but worry about cancer recurrence. This leaves women constantly questioning whether they should continue ET and work through the side-effects or stop and risk the return of cancer. This is akin to a horned dilemma—women face two equally problematic options. Those who choose to stop ET reconcile their worries by accepting their life’s impermanence.

### Theoretical category 1: guided by the doctors – accepting the long-term prescription

Women with breast cancer are initially treated with surgery, radiotherapy and/or chemotherapy, depending on their diagnosis. After this, those eligible are prescribed tamoxifen or an aromatase inhibitor as ET to take long term, essentially to prevent recurrence. The first step in the long-term management of HR-positive breast cancer is therefore the prescription of the ET (see Table 1). The causal condition, the actions/interactions, the consequences and the mediating factors for this first category are detailed here. For all the extracts referenced within the text below, see Online Resource 10.

**Table 1:** The paradigm model for the category ‘Guided by the doctors: accepting the long-term prescription’, explains the complex events when women are first prescribed ET for the long term management of their breast cancer

Context: Completing the acute stage of treatment for breast cancer *The care of women being treated for breast cancer, in the UK guided by the National Institute for Health and Care Excellence (NICE), involves treating women with chemotherapy, radiotherapy or surgery at the acute stage and with long-term treatment with a hormonal drug given as appropriate*				
Trust in their health care provider	Causal conditions	Actions/interactions	Consequences	Worries and expectations
	Transitioning into a new stage of breast cancer treatment Being overwhelmed by information provided all at once Fear of cancer recurrence Fear of possible side-effects of the new treatment Lack of specific information and uncertainty about the medication (*necessity, efficacy, safety and mechanism of action*) Feeling vulnerable The memory of difficult experiences during the initial stage of the treatment (*at a personal level, professional level or emotional level*)	Having a consultation about the medication where it is prescribed Accepting or deferring the treatment *(dependent on* e.g. *trusting their health care provider advice, awareness of the necessity of the medication, the level of stability in family and social life, emotional stability and support, desire to continue living cancer free, comorbidities, need of normalcy)* Women taking care of themselves Women looking for information elsewhere (*through specialized websites, specialized forums or from other patients*)	Going along with the hormonal prescription Delaying the hormonal treatment Transitioning into the long-term treatment phase with ease (*trusting the treatment and finding the necessary support*) Having difficulties transitioning into the long-term treatment phase Being well informed by receiving the correct information (*not looking for information in the wrong places and correcting misconceptions about the treatment*) Being wrongly informed about the medication (*side-effects, mechanism of action, efficacy and safety*) Less hospital visits (*less communication with health care providers*) Guided by the doctors: accepting the long-term prescription	
Knowledge about the treatment				

#### Causal conditions

Women are asked to start ET immediately but transitioning to this is not straightforward. Some can feel overwhelmed by the information being given to them all at once (Online Resource 10, extract 1), while having to manage the fear of recurrence and worry about the possible side-effects of the new medication (extracts 2 and 3). Altogether, the data highlight women’s need for more specific information about their condition and its treatment at this stage, fundamentally because they are uncertain about the need for further treatment. Women can feel vulnerable and emotionally unsettled especially as the acute stage of treatment would have been difficult on a physical and/or psychological level. Some women need a gap before immediately transitioning to a new treatment stage (extract 4).

#### Actions/interactions

Accepting ET or delaying the start is influenced by a range of factors, for example, trusting the healthcare provider, having stable and supportive family or friends, own emotional and psychological stability, the presence of comorbidities and a desire to continue living cancer free (extract 4). At this juncture, women are also obliged to take on a greater level of responsibility for their own care. This results in fewer hospital visits, and less frequent communication with healthcare providers, with some finding this shift difficult to adjust to. Some look elsewhere for information they have not been able to obtain from the health professionals. Women want, for example, the newest information about their treatment, the latest clinical trials, published studies about breast cancer and information on the medication they are about to use (extract 6).

#### Consequences

The main consequence here is acceptance of the first prescription, albeit for some with a delay. While women who view ET as a necessity for the overall success of their treatment proceed to long-term management with relative ease, others defer the start until, for example, they regain personal control over their life (extract 7). Women who seek information about their condition and treatment from other sources find this on specialist websites, forums and through other patients; the risk is being misinformed about the medication. There is a notable transition on starting long-term treatment. Those who feel well-informed report finding it easier to adapt, accepting the treatment and taking care of themselves (extract 8).

#### Mediating factors

Women’s trust in their healthcare provider, their knowledge about the treatment and worries and expectations facilitate or constrain the process of accepting the ET.

### Category 2: balancing priorities – adhering to the long-term treatment

ET should be taken for 5 years or more, so when treatment begins, the onus is on the woman to adhere to the regimen by taking the medication by mouth every day. Certainly women’s views about their treatment and its side-effects influence their adherence, but these can change during the treatment period (see Table 2).

**Table 2:** The paradigm model for the category ‘Balancing priorities: adhering to the long-term treatment’, explains the complex events when women are managing their ET in breast cancer survivorship

Context: Accepting a prescription for adjuvant endocrine therapy *Women are prescribed tamoxifen or aromatase inhibitors as adjuvant therapy after surgery, radiation or chemotherapy for breast cancer. Guidelines recommend the use of tamoxifen in pre- and post-menopausal women for 5 years and could be extended more than that if needed. Also, the extended use of aromatase inhibitors after the initial 5 years after diagnoses has been encouraged in post-menopausal women*				
Ability to adapt to the side-effects of the treatment	Causal conditions	Actions/interactions	Consequences	Knowledge about the treatment
	Trust and belief in the treatment and its necessity versus fear of treatment and its side-effects Wanting to continue living cancer free (realizing necessity of the treatment) and fearing cancer recurrence (anticipating regret) Receiving correct information about the treatment and side-effects in advance Need for knowledge vs preference for not knowing (psychological burden) Severity of side-effects experienced or feared (e.g. *menopausal or psychological)* Ease of access and availability of professional support and perceived their trustworthiness Wanting support from family, friends, co-workers and other patients. Obligations to family to get well and owing it to others to live Expense of the medications (insurance issues)	Incorporating medication into routine and watching for changes in usual routine Looking for appropriate support from specialists, GPs, nurses, pharmacists, support groups, family and friends Looking for other sources of information Trying to manage the side-effects Experimenting with alternative medicine Discuss the possibility of changing the hormone therapy medication Modifying life to adapt to the treatment and its side-effects *(*e.g. *quitting work due to lack of energy, downsizing, changing other routines such as sport/exercise, social activities, travelling, housework and frequency of sexual intercourse, taking up healthier eating habits)* The use of coping mechanisms to ease the experience (e.g. active coping and self-motivation, seeking physical and emotional support, maintaining a positive attitude, meditating, acceptance, humour	Adhering to the treatment despite being surprised by the challenges and the severity of the side-effects (i.e. finding adherence to be more difficult than originally thought) Forgetting to take the treatment as prescribed occasionally or taking a drug holiday to manage side-effects Committing to finishing the whole duration of the treatment Putting up with side-effects of the treatment Restricting social activities Side-effects of the treatment, old age and other medications get entangled Cancer and feeling ill linger throughout the treatment Balancing priorities: adhering to the long-term treatment	
Support received throughout the treatment				

#### Causal conditions

Some women believe in the preventative potency of their medication, despite the prospect of side-effects, deciding therefore to adhere at all costs. Others adhere even if not fully convinced, to prevent regret (extract 9). Knowledge about the treatment and its side-effects is an important factor at the start, but women are also surprised by the severity of the side-effects when they experience them, expressing that better information in advance might have better prepared them—others are taken aback despite having been pre-warned. Healthcare providers are criticized for not fully conveying the side-effects in advance (extract 10). Women also worry specifically about the prospect of hitherto unknown long-term effects, questioning the balance of risk versus benefit and whether persistence is worthwhile. Paradoxically, while most women need information to better adhere, too much knowledge can create an additional psychological burden (extract 11). A range of side-effects are mentioned in the data from physical to psychological (extract 12).

A strong factor is the availability and ease of access to professional support. Lack of access to cancer specialists at this stage leaves women feeling unsupported, having to decode experiences on their own. Some like consulting their GP while others do not, believing GPs lack the knowledge and/or desire to deal with breast-cancer-related issues. Pharmacists might not be a natural point of contact either (extract 13). Those who have a good relationship with healthcare providers want to adhere to their advice; not seeing the same physician during visits can thus be problematic (extract 14). Support from other people including family members, friends and co-workers is also important. Some especially benefit from contacting other patients, who will better understand their predicament. Women who have a good relationship with loved ones, receiving their support and encouragement, report adhering to their treatment, while those lacking this support report difficulties managing treatment and side-effects. The presence of important others is a strong motivation to stay well (extract 15), and women who feel they owe it to others to continue living, adhere to treatment (extract 16). A barrier to medication taking relates to costs and health insurance in certain cases which women have to overcome (extract 17).

#### Actions/interactions

Women who want to take their medication change their habits or incorporate their medication in their daily routine (extract 18). With the need for support being an important causal condition, women make a point of seeking the help and encouragement appropriate for them (extract 19). Women look to their healthcare providers for specific information, but soon turn to other sources if not satisfied with the answers given. While many look for specific information, some simply want reassurance (extract 20). Women experiencing side-effects look for ways to manage them, with varying levels of success. Some take additional medications while others are advised to change their routine to better adapt to treatment. Some report experimenting with alternative medicine. Where side-effects are severe and women are unable to manage them, their treatment might be switched to another hormonal medicine (extract 21). A range of life changes might also help manage the treatment side-effects. Women report specific coping techniques too that ease the experience (extract 22).

#### Consequences

Despite the difficulties, women attempt to adhere to their treatment plan, keeping in sight the end of treatment, committing if only to avoid future regrets. On occasions, some feel that skipping doses would be harmless, especially when side-effects become severe (extract 23). Women report surprise at the severity of side-effects which can even stop their social activities (extract 24). Women who take other medication or are older report difficulty distinguishing the exact cause of their symptoms, the different factors becoming entangled (extract 25). Sometimes women forget if they have taken their day’s dose which happens especially if there is a change in their routine (extract 26). The downside is that for some, the long-term nature of ET allows the presence of breast cancer to linger on, even if just at a psychological level (extract 27).

#### Mediating factors

Women’s ability to adapt to the side-effects of treatment, receiving support throughout the treatment, and knowledge about the treatment facilitate or constrain the process of adhering long-term to the ET.

### Category 3: taking a chance – stopping the treatment early

Women are expected to adhere to their ET for a duration of at least 5 years. However, some decide to stop early having taken the medication for a shorter time (see Table 3).

**Table 3:** The paradigm model for the category ‘Taking a chance: stopping the treatment early’, explains the complex events when women are deciding to stop their treatment of ET ahead of time

Context: Adhering to the medication and experiencing the side-effects *After starting the treatment and committing to adhere, women start to experience the medication side-effects, which is unexpected or more severe than they had imagined or expected*				
Quality of life taking precedence over longevity of life	Causal conditions	Actions/interactions	Consequences	Continuous search of normalcy
	Severity of the treatment sever side-effects Poor quality of life No trust in the treatment (i.e. negative perceptions of the treatment) Fear of the possible side-effects Being given the choice to stop the treatment by the healthcare provider Faith and religion A sense that existing adherence has already conferred therapeutic benefit Lack of support during the treatment Lack of trust in the healthcare providers and the medical system	Communication with health care providers and deciding to stop the treatment Stopping the treatment without communicating with anyone	Stopping the treatment early Accepting that death is not the worst option Better quality of life Regaining control Having a sense of normalcy Taking a chance: stopping the treatment early	
Beliefs about the treatment necessity				

#### Causal conditions

The most quoted reason for stopping treatment early is the severity of the side-effects (extract 28), which can greatly impact women’s quality of life, compelling them to reflect on their priorities. Living longer stops being the main concern with a more contented life free of side-effects preferred even if it is shorter. For some, initial doubts about the medication grow to overshadow any potential for benefit, especially if faced with obstacles or encouraged by others to give up. Some report their healthcare provider giving them a clear choice to stop, with others reporting their ambivalence. Some cite lack of support during the treatment as the reason for stopping prematurely, while others do it because they stop trusting their healthcare provider or the entire healthcare machinery. Some also reason that having taken the medication for a while would have conferred sufficient cover, negating the need for further doses (extract 29). Belief in the healing power of god, versus modern medicine, was also cited in the data, albeit less frequently (extract 30).

#### Actions/interactions

The decision to stop treatment early might be made jointly by the woman and her healthcare provider, or she might decide to stop ET without discussing it formally (extract 31). Ultimately, the action here is to permanently stop taking the ET.

#### Consequences

Feeling empowered to stop, women believe that taking the treatment is no longer necessary and does not justify the side-effects. This is not a choice they would have anticipated at the start but arises because of their experience with the medication, revealing to them that living longer is not what matters anymore (extract 32). Stopping treatment early leaves women feeling better in themselves, happier, more energetic and like ‘their old selves’. Their quality of life improves greatly and their sense of normalcy returns (extract 33).

#### Mediating factors

Quality of life taking precedence over longevity of life, beliefs about the treatment necessity and a continuous search of normalcy facilitate or constrain the process of stopping ET early.

## Discussion

Previous studies have documented the extent of non-adherence to ET in breast cancer survivors despite the documented risks [25–29]. Much of that literature considers medication adherence from a biomedical standpoint, seeing non-adherence as a ‘challenge’ that needs to be tackled, for example, with the use of an ‘intervention’ [14]. In contrast, this paper focussed on collating the experiences of women prescribed ET to help explain non-adherence and discontinuation using, as much as possible, an ‘insider’ perspective. The rate of publication of qualitative studies (which largely look for the insider perspective) in this area has increased sharply, with 17 studies of the 24 included in this review having been published in the last 5 years. However, to the authors’ knowledge, this is the first to synthesize the qualitative literature and develop a theoretical understanding of women’s non-adherence to long-term ET for breast cancer using GT. The theory developed helps explain why, having committed to taking tamoxifen or an aromatase inhibitor, adherence decreases over time, and importantly, it shows the decision to cease treatment early as an active choice that is made with a credible rationale. Health professionals can use these findings to support women via client-centred approaches.

Our study illustrates the importance of knowledge and support to women at the different stages of the treatment. It suggests that empowering women with knowledge about the treatment and setting appropriate expectations beforehand, especially about the side-effects, could help improve their experience of taking ET. An educational tool for use throughout the treatment, for example, could help probe for and address a range of issues that might not otherwise be addressed. Specifically, knowledge about the treatment itself, why it is needed and the range of side-effects that might be encountered could be discussed and explored. The authors are in the process of developing such a tool, using pictograms and icons to visually represent each of the three paradigm models, with the view to helping women reflect on specific topics before, during and after medical consultations. For example, visually showing the range of possible side-effects, and the range of coping strategies, could help women prepare for the side-effects in advance of treatment start. The shift in responsibility as women begin taking ET and the dwindling professional support available to them has been recognized elsewhere [30] and is worth highlighting. This is especially important when access to specialists and oncologists is withdrawn and other healthcare professionals are seen by the women either to lack expertise or the interest to provide support. A learning tool to support the knowledge of non-specialist health professionals might also prove helpful to women.

The current study is in coherence with the Necessity-Concern Framework [31], which relates specifically to medication adherence and proposes that this behaviour is linked to the balance of treatment concerns against beliefs about treatment necessity. This ‘weighing up’ is in essence what breast cancer survivors do according to the findings of the current study. Women who think of adhering to ET as a Hobson’s choice believe that the treatment is necessary and that adhering to it will prevent cancer recurrence, no matter the severity of side-effects. They are unlikely to entertain the idea of stopping the treatment and try instead to take their medication exactly as prescribed on a daily basis. On the other hand, those with weaker beliefs about treatment necessity who experience the side-effects, start accumulating concerns about the side-effects, leaving them with the difficulty of the horned dilemma. This congruence between the core category and the Necessity-Concern Framework gives credibility to the current study. The findings of this study are particularly useful as, unlike the generic nature of the Necessity-Concern Framework, the complex and dynamic models show the detailed beliefs and experiences of women on ET.

A limitation of this study is the authors’ lack of access to the original interview transcripts with the analysis built on the quotes extracted by the original authors and their respective interpretations. Nonetheless, the model was based on 801 quotes, obtained from 24 studies to reflect the experiences of 610 survivors of breast cancer. The authors believe that this provided a sufficient basis to develop a theoretical understanding within the context of a qualitative meta-synthesis. As future work, an interview study planned by the current authors and based on the categories in this paper should allow a further examination of the theory for currency in a UK setting. As it stands, the current model provides a basis for better informing survivors of breast cancer and health professionals too about the challenges of medication taking before and during the treatment process.

## Conclusion

The core category explained the difficulties women face with the initial decision to accept long-term ET and then the everyday challenge of continuing with the treatment or stopping it prematurely. Designing an educational tool to inform survivors and health care providers alike about the challenges for women on ET could potentially improve other women’s experience on the treatment and in turn their adherence.

## Electronic supplementary material

Online Resource 1 The search history for Pubmed database showing the queries constructed and the final yield (PDF 100 kb).

Online Resource 2 The search history for the Cumulative Index to Nursing and Allied Health Literature (CINAHL) database showing the queries constructed and the final yield (PDF 96 kb).

Online Resource 3 The search history for the Web of Science database showing the queries constructed and the final yield (PDF 96 kb).

Online Resource 4 The search history for the PsycINFO database showing the queries constructed and the final yield (PDF 96 kb).

Online Resource 5 The search history for the Wiley database showing the queries constructed and the final yield (PDF 95 kb).

Online Resource 6 The search history for the Proquest database showing the queries constructed and the final yield (PDF 96 kb).

Online Resource 7 A summary of the 24 studies included in the review (PDF 186 kb).

Online Resource 8 Quality assessment of the studies included in the review based on Hawker et al. (PDF 155 kb).

Online Resource 9 Table illustrating compliance with the reporting guideline for qualitative systematic reviews: Enhancing transparency in reporting the synthesis of qualitative research: ENTREQ (PDF 119 kb).

Online Resource 10 Quotation extracts as illustrative material (PDF 120 kb).
