# Supplementary material for: Adherence to adjuvant endocrine therapy among breast cancer survivors: a systematic review and meta-synthesis of the qualitative literature using grounded theory
Source: Support Care Cancer. 2020 Jun 29;28(11):5075–84. doi: 10.1007/s00520-020-05585-9 (PMC7546985; doi:10.1007/s00520-020-05585-9)
Supplement: Supplementary file 1 — The search history for Pubmed database showing the queries constructed and the final yield (PDF 100 kb). [file 520_2020_5585_MOESM1_ESM.pdf]

Adherence to adjuvant endocrine therapy among breast cancer survivors: a systematic review and meta-synthesis of the qualitative literature using grounded theory

Supportive Care in Cancer

Othman AlOmeir*; Nilesh Patel; Parastou Donyai
* Corresponding author: Othman AlOmeir, Department of Pharmacy, University of Reading, PO Box 226, Whiteknights, Reading, Berkshire RG6 6AP, UK. E-mail: o.k.o.alomeir@pgr.reading.ac.uk; Telephone number: +44 (0)118 378 4704

Online Resource 1. The search history for Pubmed database showing the queries constructed and the final yield

Search	Query	Items found
**#1**	Search ((((((((((((((((((((((((((((((Cancer) OR Tumor) OR Tumors) OR Chemotherapy) OR Oncology) OR Antineoplastic) OR Antineoplastics) OR Antineoplastic Drugs) OR Antitumor Drugs) OR Neoplasm) OR Neoplasia) OR Antineoplastic Agents) OR Anticancer Agents) OR Antitumor Agents) OR Cancer Chemotherapy Agents) OR Cancer Chemotherapy Drugs) OR Chemotherapeutic Anticancer Agents) OR Chemotherapeutic Anticancer Drug) OR Anti-Carcinogenic Agents) OR Anticarcinogenic Agents) OR Anti-Carcinogenic Drugs) OR Anticarcinogenic Drugs) OR Anticarcinogens) OR Cancer Therapy) OR Cancer Pharmacologic Therapy) OR Cancer Pharmacotherapy))) OR Tumour) OR Tumours) OR Antitumour Drugs) OR Antitumour Agents)	7021305
**#2**	Search (((((((((((((((((((Adherence) OR Compliance) OR Nonadherence) OR Non-adherence) OR Noncompliance) OR Non-compliance) OR Medication Adherence) OR Medication compliance) OR Medication Non-adherence) OR Medication Non-compliance) OR Medication Nonadherence) OR Medication Noncompliance) OR Medication Persistence) OR Patient Adherence) OR Patient Compliance) OR Patient Non-adherence) OR Patient Non-compliance) OR Patient Nonadherence) OR Patient Noncompliance) OR Patient Cooperation)	293498
**#3**	Search (((((((((((((((((Oral) OR Oral Administration) OR Administration, Oral) OR Administration, Oral Drug) OR Drug Administration, Oral) OR Oral Drug Administration) OR Oral Medicine) OR Medicine, Oral) OR Oral Medication) OR Medication, Oral) OR Tablets) OR Tamoxifen OR anastrozole OR exemestane OR letrozole OR hormonal therapy OR Hormone therapy OR Antihormonal therapy))) OR adjuvant endocrine therapy) OR Aromatase Inhibitor))	1685394
**#4**	Search (qualitative) OR qualitative research	219673
**#5**	Search (((**#1**) AND **#2**) AND **#3**) AND **#4)**	251
