# Supplementary material for: Adherence to adjuvant endocrine therapy among breast cancer survivors: a systematic review and meta-synthesis of the qualitative literature using grounded theory
Source: Support Care Cancer. 2020 Jun 29;28(11):5075–84. doi: 10.1007/s00520-020-05585-9 (PMC7546985; doi:10.1007/s00520-020-05585-9)
Supplement: Supplementary file 2 — The search history for the Cumulative Index to Nursing and Allied Health Literature (CINAHL) database showing the queries constructed and the final yield (PDF 96 kb). [file 520_2020_5585_MOESM2_ESM.pdf]

**Adherence to adjuvant endocrine therapy among breast cancer survivors: a systematic review and meta-synthesis of the qualitative literature using grounded theory**

*Supportive Care in Cancer*

Othman AlOmeir*; Nilesh Patel; Parastou Donyai
* Corresponding author: Othman AlOmeir, Department of Pharmacy, University of Reading, PO Box 226, Whiteknights, Reading, Berkshire RG6 6AP, UK. E-mail: o.k.o.alomeir@pgr.reading.ac.uk; Telephone number: +44 (0)118 378 4704

## Online Resource 2. The search history for the Cumulative Index to Nursing and Allied Health Literature (CINAHL) database showing the queries constructed and the final yield

| Search | Query | Search options | Items found |
|---|---|---|---|
| **S1** | Cancer OR tumor OR chemotherapy OR oncology OR neoplasia OR neoplasm | No limiters Apply all related words | 3572944 |
| **S2** | Adherence OR Compliance OR nonadherence OR noncompliance | No limiters Apply all related words | 278895 |
| **S3** | Oral | No limiters Apply all related words | 652461 |
| **S4** | Qualitative | No limiters Apply all related words | 175291 |
| **S5** | S1 AND S2 AND S3 AND S4 | No limiters | 28 |
