# Supplementary material for: Adherence to adjuvant endocrine therapy among breast cancer survivors: a systematic review and meta-synthesis of the qualitative literature using grounded theory
Source: Support Care Cancer. 2020 Jun 29;28(11):5075–84. doi: 10.1007/s00520-020-05585-9 (PMC7546985; doi:10.1007/s00520-020-05585-9)
Supplement: Supplementary file 4 — The search history for the PsycINFO database showing the queries constructed and the final yield (PDF 96 kb). [file 520_2020_5585_MOESM4_ESM.pdf]

**Adherence to adjuvant endocrine therapy among breast cancer survivors: a systematic review and meta-synthesis of the qualitative literature using grounded theory**

*Supportive Care in Cancer*

Othman AlOmeir*; Nilesh Patel; Parastou Donyai
* Corresponding author: Othman AlOmeir, Department of Pharmacy, University of Reading, PO Box 226, Whiteknights, Reading, Berkshire RG6 6AP, UK. E-mail: o.k.o.alomeir@pgr.reading.ac.uk; Telephone number: +44 (0)118 378 4704

## Online Resource 4. The search history for the PsycINFO database showing the queries constructed and the final yield

| Search | Query | Items found |
|---|---|---|
| 1 | (Cancer or Tumor or Tumour or Tumors or Tumours or Chemotherapy or Oncology or Antineoplastic or Antineoplastics or Antineoplastic Drugs or Antitumor Drugs or Antitumour Drugs or Neoplasm or Neoplasia or Antineoplastic Agents or Anticancer Agents or Antitumor Agents or Antitumour Agents or Cancer Chemotherapy Agents or Cancer Chemotherapy Drugs or Chemotherapeutic Anticancer Agents or Chemotherapeutic Anticancer Drug or Anti-Carcinogenic Agents or Anticarcinogenic Agents or Anti-Carcinogenic Drugs or Anticarcinogenic Drugs or Anticarcinogens or Cancer Therapy or Cancer Pharmacologic Therapy or Cancer Pharmacotherapy) | 74588 |
| 2 | (Adherence or Compliance or Nonadherence or Non-adherence or Noncompliance or Non-compliance or Medication Adherence or Medication Compliance or Medication Non-adherence or Medication Non-compliance or Medication Nonadherence or Medication Noncompliance or Medication Persistence or Patient Adherence or Patient Compliance or Patient Non-adherence or Patient Non-compliance or Patient Nonadherence or Patient Noncompliance or Patient Cooperation) | 54931 |
| 3 | (Oral or Oral Administration or Administration, Oral or Administration, Oral Drug or Drug Administration, Oral or Oral Drug Administration or Oral Medicine or Medicine, Oral or Oral Medication or Medication, Oral or Tablets) | 62288 |
| 4 | (Qualitative or Qualitative Research) | 153868 |
| 5 | 1 and 2 and 3 and 4 | 8 |
