# Supplementary material for: Adherence to adjuvant endocrine therapy among breast cancer survivors: a systematic review and meta-synthesis of the qualitative literature using grounded theory
Source: Support Care Cancer. 2020 Jun 29;28(11):5075–84. doi: 10.1007/s00520-020-05585-9 (PMC7546985; doi:10.1007/s00520-020-05585-9)
Supplement: Supplementary file 7 — A summary of the 24 studies included in the review (PDF 186 kb). [file 520_2020_5585_MOESM7_ESM.pdf]

Adherence to adjuvant endocrine therapy among breast cancer survivors: a systematic review and meta-synthesis of the qualitative literature using grounded theory
Supportive Care in Cancer
Othman AlOmeir*; Nilesh Patel; Parastou Donyai
* Corresponding author: Othman AlOmeir, Department of Pharmacy, University of Reading, PO Box 226, Whiteknights, Reading, Berkshire RG6 6AP, UK. E-mail: o.k.o.alomeir@pgr.reading.ac.uk; Telephone number: +44 (0)118 378 4704

Online Resource 7: A summary of the 24 studies included in the review

Study No.	Study	Study Location	Participant numbers	Study Objectives	Data Collection	Data Analysis	Results
1	**Harrow et al. 2014 [1]**	Scotland	30 women	To investigate the experience of women taking adjuvant endocrine therapy and what might influence their adherence	Semi-structured face to face interviews	Constant comparison method	**The eight themes identified in the study are:**<br>Lifeline to being cancer free<br>Doctor knows best<br>Remembering not to forget—got the routine<br>It's a religion<br>Living with the side effects—I'm still alive<br>Keeping it to themselves —everyone's different<br>No one's ever asked if I'm still taking it<br>Appropriate expertise
2	**Wei et al. 2017 [2]**	Shanghai, China	17 Participants	To explore the differing perspectives of patients and providers and their assessment of supportive care needs in breast cancer patients receiving oral chemotherapy.	A semi-structured interview followed by one or two in-depth interviews	Qualitative content analysis	**The five themes identified in the study are:**<br>The patient–provider discordance in assessing information needs<br>The patient–provider discordance in assessing communication needs<br>The patient–provider discordance in assessing social support needs<br>The patient–provider concordance in assessing needs of symptom and side-effects management<br>The patient–provider discordance in assessing other needs
3	**Van Londen et al. 2014 [3]**	Pittsburgh, USA	14 women	To conduct an investigation of women's experiences related to taking AET and managing AET-related symptoms.	4 focus groups with a semi-structured interview guide	Qualitative content analysis	**The five themes identified in the study are:**<br>Adjuvant Endocrine Therapy as a non-decision initially<br>Unanticipated symptoms<br>Difficulty making sense of symptoms<br>Frustration in managing symptoms<br>Weighing pros and cons of ongoing treatment

4	**Wells et al. 2016 [4]**	South-eastern, USA	25 women	To evaluate the barriers and facilitators to taking anti-hormonal medications among medically and historically underserved breast cancer survivors within the first 5 years post chemotherapy, radiation, and/or surgery.	Semi-structured interviews	Qualitative content analysis	**The two themes identified in the study are:**<br>Facilitators of medication adherence<br>Barriers to medication adherence
5	**Iacorossi et al. 2016 [5]**	Rome, Italy	27 women	To explore the experiences of adherence to endocrine therapy in women with breast cancer and their perceptions of the challenges they face in adhering to their medication prescribed	Face to face semi-structured interviews	Framework analysis in accordance with Ritchie and Spencer's approach.	**The seven themes identified in the study are:**<br>The different faces of adherence<br>Fear of the drug<br>Adherence stimulates the balance of the experience of illness<br>Adherence influences the future of disease<br>Adherence requires paying attention to the person<br>Knowledge seeking<br>Forgetfulness activates the search for functional strategies
6	**Bourmaud et al. 2016 [6]**	France	17 women	To develop and test the feasibility of a tailored therapeutic educational program, with the aim of improving adherence to oral endocrine adjuvant chemotherapy in women with breast cancer.	One-on-one interviews	Mixed qualitative (Qualitative content analysis) and quantitative method	**The five themes identified in the study are:**<br>Poor knowledge of the disease prognosis and the effects of the adjuvant endocrine therapy<br>Anxiety<br>Loneliness and lack of understanding<br>Worry about the occasional but distressing nonadherence<br>Need for skills in side effect management
7	**Flanagan et al. 2016 [7]**	Boston, USA	14 women	To describe the experience of women with oestrogen receptor-positive breast cancer who are initiating oral adjuvant therapy and to determine what they describe as facilitating and/or hindering this experience.	Semi-structured interviews	Hermeneutic phenomenological design	**The five themes identified in the study are:**<br>Feeling overwhelmed and abandoned despite highly skilled medical care<br>Processing the trauma and putting it in perspective<br>Keeping up the facade while feeling vulnerable<br>Needing to connect cautiously<br>Moving toward healing and being aware
8	**Rust and Davis 2011 [8]**	USA	24 women	To explore the issues of health literacy and medication adherence among underserved breast cancer survivors.	2 focus groups with open ended questions	Grounded theory	**The four themes identified in the study are:**<br>Inequality of access to health information<br>Acquisition of medication information<br>Medication usage and adherence<br>Barriers to access to medications

**9**	**Pellegrini et al. 2010 [9]**	South eastern France	34 women	To determine how their perceptions of the treatment and their experience of side-effects contributed to their adherence to the treatment.	Semi-structured interviews	Grounded theory	**The three themes identified in the study are:**<br>Conflicting representations about the hormonal/anti-hormonal effects of tamoxifen<br>The need for clarification about the causes of the perceived menopausal symptoms<br>Making sense of a paradoxical situation
**10**	**Adams et al. 2017 [10]**	Alabama, USA	15 women	To explore the survivorship experience, concerns, and needs of AA-BCS in rural Alabama with the goal of modifying an existing evidence-based breast cancer survivorship intervention for cultural relevance to rural AA-BCS.	Focus groups and Individual interviews	Qualitative content analysis	**The four themes identified in the study are:**<br>Cancer is a secret<br>Perish with lack of knowledge<br>Start with a good prayer life<br>Limited survivorship support and education
**11**	**Wickersham et al. 2012 [11]**	Pittsburgh, USA	12 women	To describe the medication-taking experiences of postmenopausal women with early stage breast cancer who were receiving the oral hormonal agent, anastrozole.	Semi-structured interviews	Qualitative content analysis	**The three themes identified in the study are:**<br>Perceptions about Anastrozole—“What I Think”: Keeping the Boogie Man Away<br>Side Effects and Side Effect Severity—“How It Makes Me Feel”: Being Thrown Back into Menopause<br>Day-to-Day Self-Management—“What I Do”: Doing It Yourself
**12**	**Farias et al. 2017 [12]**	Los Angeles, California and Huston, Texas, USA	22 women	To better understand how physicians communicate with breast cancer patients about adjuvant endocrine therapy (AET), we explored, from the breast cancer patient’s perspective, dimensions of the patient-provider communication among women who were on active AET treatment.	Semi-structured interviews	Qualitative principles of inductive reasoning	**The four themes identified in the study are:**<br>Information exchange between physicians and patients about AET treatment<br>Decision-making to take and continue AET treatment<br>Enabling patient self-management and monitoring potential side effects<br>Emotional support
**13**	**Wouters et al. 2013 [13]**	Netherlands	37 women	To identify the nature of the experiences and beliefs of women treated with endocrine therapy in an attempt to find potential determinants of non-adherence.	Online focus groups and Individual interviews	Hierarchical cluster analysis	**The nine themes identified in the study are:**<br>Conversations with your physician / nurse practitioner<br>Perceived Support<br>Knowledge of endocrine therapy<br>Use of Medicine<br>Efficacy

							Adverse effects & events<br>Health<br>Coping with<br>Reflection
**14**	**Brauer et al. 2016 [14]**	Los Angeles, California, USA	27 women	To explore how survivors of early-stage breast cancer, age 65 years and older, made decisions about persisting with AIs, including specific challenges as well as attempts to manage them.	Semi-structured interviews	Grounded theory	**The ten themes identified in the study are:**<br>Context of transitional survivorship<br>Lack of AI discussion in oncology follow-up care<br>Adverse effects as barrier to normalcy<br>Disentangling adverse effects from old age<br>Disentangling adverse effects from pre-existing conditions<br>Weighing up<br>Bearing the AI<br>Avoiding additional medications<br>AI switching<br>Tipping points: physical thresholds and advances in medical research
**15**	**Cahir et al. 2015 [15]**	Ireland	31 women	To use qualitative methods to investigate influences on adjuvant hormonal therapy medication taking behaviours in women with stage I–III breast cancer.	Semi-structured interviews	Thematic analysis using Theoretical Domain Framework	**The twelve domains identified in the study are:**<br>Knowledge<br>Social influences<br>Social Identity<br>Beliefs about capabilities<br>Beliefs about consequences<br>Reinforcement<br>Intentions and goals<br>Personality<br>Emotion<br>Behaviour regulation<br>Memory, attention and decision processes<br>Environmental context and resources
**16**	**Cheng et al. 2017 [16]**	Hongkong	19 women	To reveal breast cancer survivors views and experiences of self-management in extended survivorship.	Secondary analysis of the qualitative data derived from a previous study	Qualitative content analysis	**The three themes identified in the study are:**<br>Managing health and well-being<br>Managing emotions<br>Managing roles and relationships
**17**	**Verbrugghe et al. 2017 [17]**	Belgium	31 women	To give insight into the process of non-adherence and non-persistence by	Semi-structured interviews	Grounded theory	**Factors influencing the process of non-adherence and non-persistence**

				researching influencing factors and their interrelatedness in breast cancer patients taking antihormonal therapy			Experience with the previous trajectory: the context for antihormonal therapy<br>Expectations regarding the impact of antihormonal therapy<br>Impact of the antihormonal therapy and the experience of the follow-up period<br>Perceptions of the antihormonal therapy<br>Social support<br>**The process of non-adherence and non-persistence**<br>Participants experiencing a low impact<br>Participants experiencing a high impact
**18**	**Moon et al. 2017 [18]**	London, UK	32 women	To understand women experiences of taking tamoxifen and to identify factors which may be associated with non-adherence.	Semi-structured interviews	Thematic analysis	**The three themes identified in the study are:**<br>Weighing up beliefs about the treatment<br>Living with increased risk of recurrence<br>Information and support
**19**	**Brett et al. 2018 [19]**	Oxford, UK	32 women	To explore factors that influence adherence and nonadherence to adjuvant endocrine therapy following breast cancer to inform the development of supportive interventions.	Semi-structured interviews	The framework approach	**Identified factors associated with adherence were as follows:** managing side effects<br>taking control of side effects<br>supportive relationships<br>personal influences.<br>**Identified factors associated with nonadherence were as follows:** burden of side effects<br>feeling unsupported<br>concerns about long-term adjuvant endocrine therapy use<br>regaining normality<br>Risk perception and understanding the risk
**20**	**Bluethmann et al. 2017 [20]**	Dallas, Texas, USA	30 women	To build on survey results to qualitatively explore survivors' experiences with prescribed adjuvant endocrine therapy to (a) describe appraisal and management of adjuvant endocrine therapy side effects and (b) deconstruct decisions to initiate, discontinue, or maintain adjuvant endocrine therapy.	Semi-structured interviews	Mixed qualitative (Thematic analysis) and quantitative method	**The four themes identified in the study are:**<br>Initial acceptance of the provider recommendation for adjuvant endocrine therapy<br>Variable experiences with side effects<br>Risk versus reward<br>Ability to tolerate side effects

**21**	**Humphries et al. 2018 [21]**	Quebec, Canada	43 women	To identify women's attitudinal, normative, and control beliefs regarding AET adherence that could be targeted by an intervention offered in the community pharmacy setting.	Focus groups and Individual interviews	Thematic analysis based on constructs derived from the theory of planned behaviour	**The four themes identified in the study are:**<br>Attitudinal Beliefs<br>Normative Beliefs<br>Control Beliefs<br>Other constructs including:<br>Perceived Risk<br>Anticipated Regret<br>Moral Standards<br>Self-Identity
**22**	**Karlsson et al. 2019 [22]**	Sweden	25 women	To provide qualitative data about women's experiences with ET after breast cancer surgery.	Focus groups	inductive content analysis	**The three themes identified in the study are:**<br>Creates discomfort<br>Promotes levels of management<br>Causes feelings of abandonment
**23**	**Lambert et al., 2018 [23]**	Canada	22 women	To explore breast cancer survivors' experiences and perspectives of AET use to describe how personal, social, and structural factors influence AET persistence.	Semi-structured interviews	Thematic analysis	**The six themes identified in the study are:**<br>Side effects<br>Personal beliefs about recurrence and medications<br>Social support<br>HCP relationship<br>Structural factors<br>Balancing quality and quantity of life
**24**	**Xu and Wang, 2019 [24]**	China	30 women	To describe the connotations of health beliefs about AET in premenopausal breast cancer survivors in Northeast China and to explore the reasons underlying bad behaviours and influential factors of AET adherence and persistence.	Semi-structured interviews	Qualitative content analysis	**The six themes identified in the study are:**<br>Cognitions and understanding regarding AET<br>Recognition of illness recurrence and metastasis<br>Behavioural clues for treatment<br>Self-efficacy for AET<br>Demographic factors underlying health beliefs<br>The influence of socio-cultural factors on health beliefs

Full references of the included studies

1. Harrow A, Dryden R, McCowan C, et al (2014) A hard pill to swallow: a qualitative study of women's experiences of adjuvant endocrine therapy for breast cancer. Bmj Open 4:e005285. https://doi.org/10.1136/bmjopen-2014-005285

2. Wei C, Nengliang Y, Yan W, et al (2017) The patient-provider discordance in patients' needs assessment: a qualitative study in breast cancer patients receiving oral chemotherapy. J Clin Nurs 26:125–132. https://doi.org/10.1111/jocn.13374

3. van Londen GJ, Donovan HS, Beckjord EB, et al (2014) Perspectives of Postmenopausal Breast Cancer Survivors on Adjuvant Endocrine Therapy-related Symptoms. Oncol Nurs Forum 41:660–668. https://doi.org/10.1188/14.ONF.660-668

4. Wells KJ, Pan TM, Vázquez-otero C, et al (2016) Barriers and facilitators to endocrine therapy adherence among underserved hormone-receptor-positive breast cancer survivors: a qualitative study. Support Care Cancer Heidelb 24:4123–4130. http://dx.doi.org/10.1007/s00520-016-3229-8

5. Iacorossi L, Gambalunga F, Fabi A, et al (2016) Adherence to Oral Administration of Endocrine Treatment in Patients With Breast Cancer: A Qualitative Study. Cancer Nurs. https://doi.org/10.1097/NCC.0000000000000452

6. Bourmaud A, Rousset V, Regnier-Denois V, et al (2016) Improving Adherence to Adjuvant Endocrine Therapy in Breast Cancer Through a Therapeutic Educational Approach: A Feasibility Study. Oncol Nurs Forum Pittsburgh 43:E94–E103. http://dx.doi.org/10.1188/16.ONF.E94-E103

7. Flanagan J, Tetler D, Winters L, et al (2016) The Experience of Initiating Oral Adjuvant Treatment for Estrogen Receptor-Positive Breast Cancer. Oncol Nurs Forum 43:E143–E152. https://doi.org/10.1188/16.ONF.E143-E152

8. Rust C, Davis C (2011) Health literacy and medication adherence in underserved African-american breast cancer survivors: a qualitative study. Soc Work Health Care 50:739–761. https://doi.org/10.1080/00981389.2011.585703

9. Pellegrini I, Sarradon-Eck A, Ben Soussan P, et al (2010) Women's perceptions and experience of adjuvant tamoxifen therapy account for their adherence: breast cancer patients' point of view. Psychooncology 19:472–479. https://doi.org/10.1002/pon.1593

10. Adams N, Gisiger-Camata S, Hardy CM, et al (2017) Evaluating Survivorship Experiences and Needs Among Rural African American Breast Cancer Survivors. J Cancer Educ Off J Am Assoc Cancer Educ 32:264–271. https://doi.org/10.1007/s13187-015-0937-6

11. Wickersham K, Happ MB, Bender CM (2012) "Keeping the Boogie Man Away": Medication Self-Management among Women Receiving Anastrozole Therapy. Nurs Res Pract 2012:462121. https://doi.org/10.1155/2012/462121

12. Farias AJ, Ornelas IJ, Hohl SD, et al (2017) Exploring the role of physician communication about adjuvant endocrine therapy among breast cancer patients on active treatment: a qualitative analysis. Support Care Cancer Off J Multinatl Assoc Support Care Cancer 25:75–83. https://doi.org/10.1007/s00520-016-3389-6

13. Wouters H, van Geffen ECG, Baas-Thijssen MC, et al (2013) Disentangling breast cancer patients' perceptions and experiences with regard to endocrine therapy: nature and relevance for non-adherence. Breast Edinb Scotl 22:661–666. https://doi.org/10.1016/j.breast.2013.05.005

14. Brauer ER, Ganz PA, Pieters HC (2016) "Winging It": How Older Breast Cancer Survivors Persist With Aromatase Inhibitor Treatment. J Oncol Pract 12:e991–e1000. https://doi.org/10.1200/JOP.2016.011767

15. Cahir C, Dombrowski SU, Kelly CM, et al (2015) Women's experiences of hormonal therapy for breast cancer: exploring influences on medication-taking behaviour. Support Care Cancer 23:3115–3130. https://doi.org/10.1007/s00520-015-2685-x

16. Cheng H, Sit JWH, Cheng KKF (2017) A qualitative insight into self-management experience among Chinese breast cancer survivors. Psychooncology 26:1044–1049. https://doi.org/10.1002/pon.4279

17. Verbrugghe M, Verhaeghe S, Decoene E, et al (2017) Factors influencing the process of medication (non-)adherence and (non-)persistence in breast cancer patients with adjuvant antihormonal therapy: a qualitative study. Eur J Cancer Care (Engl) 26:. https://doi.org/10.1111/ecc.12339

18. Moon Z, Moss-Morris R, Hunter MS, Hughes LD (2017) Understanding tamoxifen adherence in women with breast cancer: A qualitative study. Br J Health Psychol 22:978–997. https://doi.org/10.1111/bjhp.12266

19. Brett J, Boulton M, Fenlon D, et al (2018) Adjuvant endocrine therapy after breast cancer: a qualitative study of factors associated with adherence. Patient Prefer Adherence 12:291–300. https://doi.org/10.2147/PPA.S145784

20. Bluethmann SM, Murphy CC, Tiro JA, et al (2017) Deconstructing Decisions to Initiate, Maintain, or Discontinue Adjuvant Endocrine Therapy in Breast Cancer Survivors: A Mixed-Methods Study. Oncol Nurs Forum 44:E101–E110. https://doi.org/10.1188/17.ONF.E101-E110

21. Humphries B, Collins S, Guillaumie L, et al (2018) Women's Beliefs on Early Adherence to Adjuvant Endocrine Therapy for Breast Cancer: A Theory-Based Qualitative Study to Guide the Development of Community Pharmacist Interventions. Pharmacy 6:53. https://doi.org/10.3390/pharmacy6020053

22. Karlsson SA, Wallengren C, Bagge RO, Henoch I "It is not just any pill"—Women's experiences of endocrine therapy after breast cancer surgery. Eur J Cancer Care (Engl) 0:e13009. https://doi.org/10.1111/ecc.13009

23. Lambert LK, Balneaves LG, Howard AF, et al (2018) Understanding adjuvant endocrine therapy persistence in breast Cancer survivors. BMC Cancer 18:. https://doi.org/10.1186/s12885-018-4644-7

24. Xu L, Wang A (2019) Health belief about adjuvant endocrine therapy in premenopausal breast cancer survivors: a qualitative study. Patient Prefer Adherence 13:1519–1525. https://doi.org/10.2147/PPA.S217562
