# Supplementary material for: Adherence to adjuvant endocrine therapy among breast cancer survivors: a systematic review and meta-synthesis of the qualitative literature using grounded theory
Source: Support Care Cancer. 2020 Jun 29;28(11):5075–84. doi: 10.1007/s00520-020-05585-9 (PMC7546985; doi:10.1007/s00520-020-05585-9)
Supplement: Supplementary file 8 — Quality assessment of the studies included in the review based on Hawker et al. (PDF 155 kb). [file 520_2020_5585_MOESM8_ESM.pdf]

Adherence to adjuvant endocrine therapy among breast cancer survivors: a systematic review and meta-synthesis of the qualitative literature using grounded theory
Supportive Care in Cancer
Othman AlOmeir*; Nilesh Patel; Parastou Donyai
* Corresponding author: Othman AlOmeir, Department of Pharmacy, University of Reading, PO Box 226, Whiteknights, Reading, Berkshire RG6 6AP, UK. E-mail: o.k.o.alomeir@pgr.reading.ac.uk; Telephone number: +44 (0)118 378 4704

Online Resource 8: Quality assessment of the studies included in the review based on Hawker et al.*

Study	Abstract and title (1)	Introduction and aims (2)	Method and data (3)	Sampling (4)	Data analysis (5)	Ethics and bias (6)	Findings/ results (7)	Transferability/ generalizability (8)	Implications and usefulness (9)	Total
**Harrow et al. 2014 [1]**	g	f	g	g	g	vp	g	g	g	g = 7<br>f = 1<br>vp = 1
**Wei et al. 2017 [2]**	g	p	g	p	g	p	g	g	f	g = 5<br>f = 1<br>p = 3
**Van Londen et al. 2014 [3]**	g	f	f	g	g	vp	g	g	f	g = 5<br>f = 3<br>vp = 1
**Wells et al. 2016 [4]**	g	g	f	g	f	vp	g	g	g	g = 7<br>f = 1<br>vp = 1
**Iacorossi et al. 2016 [5]**	g	g	g	g	g	g	g	g	g	g = 9
**Bourmaud et al. 2016 [6]**	g	f	f	g	g	p	g	g	f	g = 5<br>f = 3<br>p = 1
**Flanagan et al. 2016 [7]**	g	f	g	g	g	g	g	g	f	g = 7<br>f = 2
**Rust and Davis 2011 [8]**	f	g	g	g	g	vp	g	f	p	g = 5<br>f = 2<br>p = 1<br>vp = 1

**Pellegrini et al. 2010 [9]**	g	f	f	g	g	p	f	g	f	g = 4<br>f = 4<br>p = 1
**Adams et al. 2017 [10]**	g	f	g	vp	g	vp	g	g	f	g = 5<br>f = 2<br>vp = 2
**Wickersham et al. 2012 [11]**	g	f	g	g	g	p	g	g	g	g = 7<br>f = 1<br>p = 1
**Farias et al. 2017 [12]**	g	f	g	g	g	f	g	g	g	g = 7<br>f = 2
**Wouters et al. 2013 [13]**	g	g	g	g	f	f	f	f	f	g = 4<br>f = 5
**Brauer et al. 2016 [14]**	g	f	g	g	g	f	g	g	g	g = 7<br>f = 2
**Cahir et al. 2015 [15]**	g	f	g	g	g	f	g	g	g	g = 7<br>f = 2
**Cheng et al. 2017 [16]**	g	f	f	f	g	f	f	f	f	g = 2<br>f = 7
**Verbrugghe et al. 2017 [17]**	f	f	g	g	g	g	g	g	g	g = 7<br>f = 2
**Moon et al. 2017 [18]**	g	p	g	f	g	f	g	f	f	g = 4<br>f = 4<br>p = 1
**Brett et al. 2018 [19]**	g	g	g	g	g	f	g	g	g	g = 8<br>f = 1
**Bluethmann et al. 2017 [20]**	g	f	g	g	f	p	g	f	f	g = 4<br>f = 4<br>p = 1
**Humphries et al. 2018 [21]**	f	f	g	g	g	p	g	g	g	g = 6<br>f = 2<br>p = 1
**Karlsson et al. 2019 [22]**	g	f	g	g	g	g	g	g	f	g=7<br>f=2
**Lambert et al., 2018 [23]**	g	f	g	g	g	g	g	g	g	g=8<br>f=1

**Xu and Wang, 2019 [24]**	g	p	f	p	g	f	g	f	f	g=3<br>f=4<br>p=2

(1) Abstract and title: Did they provide a clear description of the study? **(2) Introduction and aims:** Was there a good background and clear statement of the aims of the research? **(3) Method and data:** Is the method appropriate and clearly explained? **(4) Sampling:** Was the sampling strategy appropriate to address the aims? **(5) Data analysis:** Was the description of the data analysis sufficiently rigorous? **(6) Ethics and bias:** Have ethical issues been addressed, and what has necessary ethical approval gained? Has the relationship between research and participants been adequately considered? **(7) Results:** Is there a clear statement of the findings? **(8) Transferability or generalizability:** Are the findings of this study transferable (generalizable) to a wider population? **(9) Implications and usefulness:** How important are these findings to policy and practice? **Criteria: g = good; f = fair, p = poor; vp = very poor.**

Full references of the included studies

1. Harrow A, Dryden R, McCowan C, et al (2014) A hard pill to swallow: a qualitative study of women's experiences of adjuvant endocrine therapy for breast cancer. Bmj Open 4:e005285. https://doi.org/10.1136/bmjopen-2014-005285

2. Wei C, Nengliang Y, Yan W, et al (2017) The patient-provider discordance in patients' needs assessment: a qualitative study in breast cancer patients receiving oral chemotherapy. J Clin Nurs 26:125–132. https://doi.org/10.1111/jocn.13374

3. van Londen GJ, Donovan HS, Beckjord EB, et al (2014) Perspectives of Postmenopausal Breast Cancer Survivors on Adjuvant Endocrine Therapy-related Symptoms. Oncol Nurs Forum 41:660–668. https://doi.org/10.1188/14.ONF.660-668

4. Wells KJ, Pan TM, Vázquez-otero C, et al (2016) Barriers and facilitators to endocrine therapy adherence among underserved hormone-receptor-positive breast cancer survivors: a qualitative study. Support Care Cancer Heidelb 24:4123–4130. http://dx.doi.org/10.1007/s00520-016-3229-8

5. Iacorossi L, Gambalunga F, Fabi A, et al (2016) Adherence to Oral Administration of Endocrine Treatment in Patients With Breast Cancer: A Qualitative Study. Cancer Nurs. https://doi.org/10.1097/NCC.0000000000000452

6. Bourmaud A, Rousset V, Regnier-Denois V, et al (2016) Improving Adherence to Adjuvant Endocrine Therapy in Breast Cancer Through a Therapeutic Educational Approach: A Feasibility Study. Oncol Nurs Forum Pittsburgh 43:E94–E103. http://dx.doi.org/10.1188/16.ONF.E94-E103

7. Flanagan J, Tetler D, Winters L, et al (2016) The Experience of Initiating Oral Adjuvant Treatment for Estrogen Receptor-Positive Breast Cancer. Oncol Nurs Forum 43:E143–E152. https://doi.org/10.1188/16.ONF.E143-E152

8. Rust C, Davis C (2011) Health literacy and medication adherence in underserved African-american breast cancer survivors: a qualitative study. Soc Work Health Care 50:739–761. https://doi.org/10.1080/00981389.2011.585703

9. Pellegrini I, Sarradon-Eck A, Ben Soussan P, et al (2010) Women's perceptions and experience of adjuvant tamoxifen therapy account for their adherence: breast cancer patients' point of view. Psychooncology 19:472–479. https://doi.org/10.1002/pon.1593

10. Adams N, Gisiger-Camata S, Hardy CM, et al (2017) Evaluating Survivorship Experiences and Needs Among Rural African American Breast Cancer Survivors. J Cancer Educ Off J Am Assoc Cancer Educ 32:264–271. https://doi.org/10.1007/s13187-015-0937-6

11. Wickersham K, Happ MB, Bender CM (2012) "Keeping the Boogie Man Away": Medication Self-Management among Women Receiving Anastrozole Therapy. Nurs Res Pract 2012:462121. https://doi.org/10.1155/2012/462121

12. Farias AJ, Ornelas IJ, Hohl SD, et al (2017) Exploring the role of physician communication about adjuvant endocrine therapy among breast cancer patients on active treatment: a qualitative analysis. Support Care Cancer Off J Multinatl Assoc Support Care Cancer 25:75–83. https://doi.org/10.1007/s00520-016-3389-6

13. Wouters H, van Geffen ECG, Baas-Thijssen MC, et al (2013) Disentangling breast cancer patients' perceptions and experiences with regard to endocrine therapy: nature and relevance for non-adherence. Breast Edinb Scotl 22:661–666. https://doi.org/10.1016/j.breast.2013.05.005

14. Brauer ER, Ganz PA, Pieters HC (2016) "Winging It": How Older Breast Cancer Survivors Persist With Aromatase Inhibitor Treatment. J Oncol Pract 12:e991–e1000. https://doi.org/10.1200/JOP.2016.011767

15. Cahir C, Dombrowski SU, Kelly CM, et al (2015) Women's experiences of hormonal therapy for breast cancer: exploring influences on medication-taking behaviour. Support Care Cancer 23:3115–3130. https://doi.org/10.1007/s00520-015-2685-x

16. Cheng H, Sit JWH, Cheng KKF (2017) A qualitative insight into self-management experience among Chinese breast cancer survivors. Psychooncology 26:1044–1049. https://doi.org/10.1002/pon.4279

17. Verbrugghe M, Verhaeghe S, Decoene E, et al (2017) Factors influencing the process of medication (non-)adherence and (non-)persistence in breast cancer patients with adjuvant antihormonal therapy: a qualitative study. Eur J Cancer Care (Engl) 26:. https://doi.org/10.1111/ecc.12339

18. Moon Z, Moss-Morris R, Hunter MS, Hughes LD (2017) Understanding tamoxifen adherence in women with breast cancer: A qualitative study. Br J Health Psychol 22:978–997. https://doi.org/10.1111/bjhp.12266

19. Brett J, Boulton M, Fenlon D, et al (2018) Adjuvant endocrine therapy after breast cancer: a qualitative study of factors associated with adherence. Patient Prefer Adherence 12:291–300. https://doi.org/10.2147/PPA.S145784

20. Bluethmann SM, Murphy CC, Tiro JA, et al (2017) Deconstructing Decisions to Initiate, Maintain, or Discontinue Adjuvant Endocrine Therapy in Breast Cancer Survivors: A Mixed-Methods Study. Oncol Nurs Forum 44:E101–E110. https://doi.org/10.1188/17.ONF.E101-E110

21. Humphries B, Collins S, Guillaumie L, et al (2018) Women's Beliefs on Early Adherence to Adjuvant Endocrine Therapy for Breast Cancer: A Theory-Based Qualitative Study to Guide the Development of Community Pharmacist Interventions. Pharmacy 6:53. https://doi.org/10.3390/pharmacy6020053

22. Karlsson SA, Wallengren C, Bagge RO, Henoch I "It is not just any pill"—Women's experiences of endocrine therapy after breast cancer surgery. Eur J Cancer Care (Engl) 0:e13009. https://doi.org/10.1111/ecc.13009

23. Lambert LK, Balneaves LG, Howard AF, et al (2018) Understanding adjuvant endocrine therapy persistence in breast Cancer survivors. BMC Cancer 18:. https://doi.org/10.1186/s12885-018-4644-7

24. Xu L, Wang A (2019) Health belief about adjuvant endocrine therapy in premenopausal breast cancer survivors: a qualitative study. Patient Prefer Adherence 13:1519–1525. https://doi.org/10.2147/PPA.S217562

* Hawker S, Payne S, Kerr C, et al (2002) Appraising the Evidence: Reviewing Disparate Data Systematically. Qual Health Res 12:1284–1299. https://doi.org/10.1177/1049732302238251
