# Supplementary material for: Adherence to adjuvant endocrine therapy among breast cancer survivors: a systematic review and meta-synthesis of the qualitative literature using grounded theory
Source: Support Care Cancer. 2020 Jun 29;28(11):5075–84. doi: 10.1007/s00520-020-05585-9 (PMC7546985; doi:10.1007/s00520-020-05585-9)
Supplement: Supplementary file 10 — Quotation extracts as illustrative material (PDF 120 kb). [file 520_2020_5585_MOESM10_ESM.pdf]

Adherence to adjuvant endocrine therapy among breast cancer survivors: a systematic review and meta-synthesis of the qualitative literature using grounded theory

Supportive Care in Cancer

Othman AlOmeir*; Nilesh Patel; Parastou Donyai
* Corresponding author: Othman AlOmeir, Department of Pharmacy, University of Reading, PO Box 226, Whiteknights, Reading, Berkshire RG6 6AP, UK. E-mail: o.k.o.alomeir@pgr.reading.ac.uk; Telephone number: +44 (0)118 378 4704

Online Resource 10: Quotation extracts as illustrative material

Extract 1, Study 2: *"I try to remember everything ... on the first day. But I can't remember anything when things happen. I really hope we can get the information gradually."*

Extract 2, Study 1: *"I see my tamoxifen as the lifeline to being cancer free."*

Extract 3, Study 6: *"The doctor frightened me so much with all the possible side effects that I asked myself, where are you going with this treatment?"*

Extract 4, Study 7: *"It's like your last infusion is ending, and you can see everyone thinks, 'OK, finally [you're] back to normal.' It feels like they are ready to pounce on you. I'm thinking, like, really, I need a minute. I've been to hell and back."*

Extract 5: Study 19: *"I must admit I take it – if they [doctors] say it's a good idea I'm very much [...] I think because my experience has been so positive with them [doctors] I've not come away doubting anything."*

Extract 6: Study 5: *"I look for information on my own...they just told me to take that drug because there was a 45% possibilities not to face other problems and nothing more! Then one compares and discusses with friends, on the Internet, and evaluates the effects that can be attributed to the drug"*

Extract 7: Study 7: *"Over this last year, I did everything I was supposed to. I have other worries I need to take care of before starting that."*

Extract 8: Study 5 (interview 20): *"Education is necessary in order to understand the importance of it all"*

Extract 9: Study 18: *"But it's like you're damned if you do and you're damned if you don't. It's that worry if you don't take it, oh god, if they find something again then I think it's because I didn't take the tamoxifen. But on the other hand it's living with all these side effects on it."*

Extract 10: Study 17: *"I think they have explained too little about side effects. They have actually minimized them, which makes them worse than I imagined them to be. Now I have to learn to deal with it after I have experienced them and this is very difficult."*

Extract 11: Study 15: *"I suppose it's mad that I've put my trust in a doctor and a drug that I probably should know more about but the problem is I would have worried myself sick if I knew all of the bad things."*

Extract 12: Study 4: *"There are days that all of you is in pain, all the body… A pain that you don't know what is hurting. And it is so horrible … you try to be still so it doesn't hurt You can't cook, you can't clean, you can't even bathe because…the pain is in all your body"*

Extract 13: Study 1: *"The relationship isn't there I wouldn't have thought about going to see the pharmacist to talk about my side-effects with any medications, I go to the GP about that."*

Extract 14: Study 12: *"He [oncologist] knew me by my name, my face. When I came in, it was like they treated you like you were a person and not just cattle coming through. He used to call me his most delicate patient."*

Extract 15: Study 24: *"My husband and two children are a motivation for me to live."*

Extract 16: Study 21: *" . . . All the effort made by everyone around us to support us. What's taking a pill? We owe them that."*

Extract 17: Study 8: *"I have had a hard time on some of my medication. The insurance don't want to pay for it The clinic won't override it and they won't give it to me If I can pay for it I pay for it"*

Extract 17: Study 21: *"I asked myself what do I do everyday of my life? At breakfast, my jar of peanut butter . . . Every morning, it is there."*

Extract 19: Study 5: *"I really needed understanding, kindness. . .I know that may be it is asking too much but we are not only 'physical' beings..."*

Extract 20: Study 2: *"Whenever I asked her if she can cure me, I actually just wanted a hope rather than an unreasonable demand"*

Extract 21: Study 1: *"I was trying to keep it going till I got to the clinic but because I felt I couldn't drive my car I stopped it because it was only about ten days before my clinic appointment But you know I mean I knew that really ten days off it wasn't going to make any difference you know in the long term, so then I got tamoxifen and I'm fine with that"*

Extract 22: Study 1: *"We're having to downsize our house so that we can accommodate the fact, because I would rather live in a smaller house costing less money, so that I have the option that if I'm still not well enough I don't have the pressure of having to go back to work"*

Extract 23: Study 18 *"I've got to the stage where sometimes I'll just give it a miss. . .I just get so fed up of taking it, I just want to give myself a break."*

Extract 24: Study 23: *"I started to withdraw from social situations. I didn't trust my body to co-operate. I missed out on quite a few things, because I was too afraid that [due to the diarrhoea] I would have to run or, change my clothes or have a shower. And make a mess in public. Emotionally, it was devastating."*

Extract 25: Study 14: *"It's very hard for me to pinpoint what's causing what because I have all of these different [health conditions]."*

Extract 26: Study 15: *"On Sunday because I'm going to church for communion and I don't have my breakfast or coffee I forget. And I come back after church and I forget."*

Extract 27: Study 21: *"It's like a trace of what we've experienced, like a passport that you always have on you."*

Extract 28: Study 3*: "You do get to a point where it just isn't worth it to fight it [staying on AET]"*

Extract 29: Study 17: *"I have been taking AHT for four years; one year will not make a difference*

Extract 30: Study 4: *"The reason I'm not taking drugs anymore is my faith. I very firmly believe that God healed me. I prayed. My church prayed for me. I did exactly what God tells us to do in the Bible and that is to go to Him and ask Him and give Him all the credit for it first, and He did heal me"*

Extract 31: Study 20: *"I took myself off the medicine. I went to my primary care physician and told him what I had done. He almost had a heart attack … and I said, I've had tamoxifen, and I've had breast cancer. I would rather have breast cancer."*

Extract 32: Study 15: *"I chose a lesser time left. I said at my age, does it matter if the cancer comes back one way or another but if I have these few years of, I don't go gallivanting or that, I like my home and I like being involved in the community, going to the club and that. Coming off the tablet has given me back that quality of life"*.

Extract 33: Study 15: *“I stopped taking it three weeks ago and I feel wonderful. I started feeling better after about a week and every week that’s gone past I'm feeling better and better, I feel like me again. I feel great.”*
